# VgrG2 of type VI secretion system 2 of *Vibrio parahaemolyticus* induces autophagy in macrophages

**DOI:** 10.3389/fmicb.2015.00168

**Published:** 2015-03-02

**Authors:** Ying Yu, Lihua Fang, Yan Zhang, Hongxia Sheng, Weihuan Fang

**Affiliations:** ^1^Institute of Health Food, Zhejiang Academy of Medical Sciences,Hangzhou, China; ^2^Institute of Preventive Veterinary Medicine and Zhejiang Provincial Key Laboratory of Preventive Veterinary Medicine, Zhejiang University,Hangzhou, China

**Keywords:** *Vibrio parahaemolyticus*, type VI secretion system, secretion system, effector proteins, autophagy

## Abstract

Type VI secretion system (T6SS) is a macromolecular transenvelope machine encoded within the genomes of several proteobacteria species. *Vibrio parahaemolyticus* contains two putative T6SS systems, VpT6SS1 and VpT6SS2, both contributing to adherence to Caco-2 and/or HeLa cells. However, it remains unknown if these systems are involved in cellular responses. In order to exclude the effects of other virulence factors known to induce cytotoxicity or autophagy, a triple deletion mutant dTTT (with deletion of *tdh*, and T3SS1 and T3SS2 structural protein genes) was used as the parent strain to construct deletion mutants of T6SS genes. The mutant dTTT-Δ*icmF2*, but not dTTT-Δ*icmF1*, reduced autophagic response upon 4 h of infection of the macrophage. Further attempt was made to search for the possible effector proteins that might be responsible for direct induction of autophagy by deletion of the genes encoding Hcp2 and VgrG2, two putative translocons of T6SS2 of *V. parahaemolyticus*. Deletion of either *hcp2* or *vgrG2* did reduce the autophagic response. However, increased LC3-II lipidation was seen only in the macrophage cells transfected with pVgrG2, but not with pHcp2. Chloroquinine treatment increased accumulation of LC3-II, suggesting that VgrG2 enhanced autophagic flux. The fact that *vgrG2* deletion led to reduced level of intracellular cAMP suggests a possible role of cAMP signaling in autophagic responses to the bacterium. We conclude that VgrG2 of *V. parahaemolyticus* induces autophagy in macrophages.

## INTRODUCTION

*Vibrio parahaemolyticus* is one of the leading causes of human foodborne gastroenteritis in China (about one-third of the foodborne outbreaks reported between 1991 and 2001) due to consumption of raw or under-cooked seafood (Liu et al., 2004). Early studies have linked gastroenteritis to the presence of thermostable direct hemolysin (TDH), TDH-related hemolysin (TRH) and two sets of type III secretion systems (T3SS1 and T3SS2), which are able to induce general cytotoxicity or enterotoxicity to host cells (Kaper et al., 1984; Park et al., 2004; Yeung and Boor, 2004; Okada et al., 2009).

Type VI secretion system is a macromolecular transenvelope machine encoded within the genomes of several proteobacterial species (Mougous et al., 2006; Pukatzki et al., 2007; Bingle et al., 2008; Bernard et al., 2010). The system contains 13–20 proteins [Intracellular multiplication Factor (IcmF)-associated homologous proteins, IAHP] coded by the gene cluster (Boyer et al., 2009). Deletion of *icmF* associated proteins did not affect expression of the translocon proteins but prevents their translocation (Pukatzki et al., 2006; Suarez et al., 2008). The T6SSs of *Vibrio cholerae*, *Pseudomonas aeruginosa*, *Aeromonas hydrophila,* and *Vibrio anguillarum* were found to participate in pathogenicity: adhesion to epithelial cells, cytotoxicity, resistance to phagocytosis, tolerance to stress sensing, and replication inside the host cells (Mougous et al., 2006; Pukatzki et al., 2006; Zheng and Leung, 2007; Suarez et al., 2008; Weber et al., 2009; Jani and Cotter, 2010). Of the two sets of putative T6SS in *V. parahaemolyticus* (VpT6SS), we found that VpT6SS1 is present in majority of clinical isolates (90.9%), but less in environmental or food isolates (25.0%) while VpT6SS2 exists in all isolates, and both systems contribute different aspects of adherence to Caco-2 and/or HeLa cells (Yu et al., 2012).

Autophagy acts as an intracellular surveillance system to monitor and trap invading pathogens and influence both the innate and adaptive immune responses (Burdette et al., 2009b; Deretic and Levine, 2009). For most intracellular bacteria, host cells use autophagy to prevent cytoplasmic replication or invasion of intracellular pathogens by engulfing the pathogens in autophagic vesicles and targeting them to lysosomes (Levine and Deretic, 2007). In extracellular bacteria like *Vibrio* spp, secreted proteins are involved in autophagy (Gutierrez et al., 2007). With *V. parahaemolyticus*, VP1680 (VopQ) and VP1659 (two secretion proteins of T3SS1) are found to induce autophagy accompanied with cytotoxicity, disruption of actin structure, and cell death (Burdette et al., 2009a; Zhou et al., 2010). By examining LC3 lipidation and EGFP-LC3 punctation in macrophages infected with *V. parahaemolyticus*, we provide the first evidence that the VgrG2, a translocon of VpT6SS2, induces autophagy.

## MATERIALS AND METHODS

### BACTERIAL STRAINS AND PLASMIDS

The bacterial strains and plasmids used in this study are listed in **Table 1**. *V. parahaemolyticus* strain HZ is a clinical isolate from the Zhejiang Provincial Center for Disease Control and Prevention, Zhejiang, China. *Escherichia coli* strains DH5α, BL21, and CC118λpir were used for general manipulation of plasmids, prokaryotic expression of proteins, and mobilization of plasmids into *V. parahaemolyticus*, respectively. The bacterial strains were grown at 37^∘^C in Luria-Bertani (LB) broth (*E. coli*) or LB broth supplemented with 3% NaCl (*V. parahaemolyticus*). LB agar supplemented with 3% NaCl, 10 μg/ml chloramphenicol, and 25 μg/ml polymyxin was used for screening mutant strains. The culture media were supplemented, where appropriate, with the following antibiotics: chloramphenicol (Cm, 10 μg/ml), ampicillin (Amp, 100 μg/ml), and kanamycin (Kan, 50 μg/ml).

**Table 1:** Bacterial strains and plasmids used in this study.

Plasmids or strains	Description	Reference or source
**Plasmids**
pMD18T	A clone vector, Amp^r^	Takara
pYAK1	A suicide vector with ori R6K *sacB*; Cm^r^	Park et al. (2004)
pET-30a	PBR322 origin, pT7, *his*-tag	Novagen
pcDNA3.1		Invitrogen
pcDNA-*egfp*		Zhu et al. (2012)
pcDNA-*egfp*-*lc3b*		Zhu et al. (2012)
pHcp2	pcDNA-*egfp* fused with *hcp2* of VpT6SS2	This study
pVgrG2	pcDNA-*egfp* fused with *vgrG2* of VpT6SS2	This study
***Escherichia coli***
CC118λpir	Λpir lysogen of CC118 Δ(*ara-leu*) *araD*Δ*lacX74 galE galK phoA20 thi-1 rpsE rpoB argE(*Am*) recA1*	Yu et al. (2012)
DH5α	F^-^ φ80*lacZΔM15* Δ*(lacZYA-argF)* U169 *deoR recA1 endA1 hsdR17 phoA supE44 λ*^-^ *thi-1 gyrA96 relA1*	Invitrogen
BL21		Novagen
***Vibrio parahaemolyticus***
HZ	Wild type (WT), clinical strain, Cm^s^	Yu et al. (2012)
dTTT	Strain HZ with in-frame deletion of *tdh, vcrD1,* and *vcrD2*	Yu et al. (2012)
Δ*icmF1*	Strain dTTT with in-frame deletion of *icmF1*	Yu et al. (2012)
Δ*icmF2*	Strain dTTT with in-frame deletion of *icmF2*	Yu et al. (2012)
Δ*icmF1/icmF2*	Strain dTTT with in-frame deletion of *icmF1* and *icmF2*	Yu et al. (2012)
Δ*hcp2*	Strain dTTT with in-frame deletion of *hcp2*	Yu et al. (2012)
Δ*vgrG2*	Strain dTTT with in-frame deletion of *vgrG2*	This study

### PLASMID CONSTRUCTION

The mammalian expression vector pcDNA-*egfp* and pcDNA-*egfp*-*lc3b* were constructed from pcDNA3.1 (Invitrogen) in our laboratory (Zhu et al., 2012). To construct pHcp2 and pVgrG2 in pcDNA3.1 background for expression of these proteins fused with GFP (**Table 1**), *egfp* was PCR-amplified from pcDNA-*egfp* by primers GFP-F/R, and genes *hcp2* and *vgrG2* were from *V. parahemolyticus* strain HZ amplified by primers *hcp2*-F/R and *vgrG2*-F/R, respectively. The *egfp*-*hcp2* and *egfp*-*vgrG2* fusion fragments were obtained by overlap PCR using primers GFP-F/*hcp2*-R and GFP-F/*vgrG2*-R, respectively, and cloned into the multiple cloning site of pcDNA3.1. The above primers are listed in **Table 2**. All constructs were confirmed by DNA sequencing.

**Table 2:** Primers used in this study.

Primers	Sequence (5^′^–3^′^)	Reference
EGFP-F	TAGGATTCGCCACCATGGTGAGCAAGGGCGA	Zhu et al. (2012)
EGFP-R	TCCTCCGCTTCCTCCCTTGTACAGCTCGTCCAT	
*hcp2*-F	GGAGGAAGCGGAGGAATGCAGTCTAATAC	This study
*hcp2*-R	GAACTCGAGTTACATTTGTTGACCT	
*vgrG2*-F	GGAGGAAGCGGAGGAATGAAAAAAGCAAGTC	This study
*vgrG2*-R	GGCCTCGAGTTAATTCAAAGAGATT	
*hcp2*-KF	CACGGATCCATGCAGTCTAATAC	This study
*hcp2*-KR	GAACTCGAGTTACATTTGTTGACCT	
*vgrG2*-KF	AAAGGATCCATGAAAAAAGCAAGTC	This study
*vgrG2*-KR	GGCCTCGAGTTAATTCAAAGAGATT	
*vgrG2*-A	AAAGGATCCTTGTACTTGGATGACCACC	This study
*vgrG2*-B	GTATCCAGAGGGAACTTAGAATGGGTAAAC	
*vgrG2*-C	GTTCCCTCTGGATACTTATATTTCCTTTTGAA	
*vgrG2*-D	ATTGCATGCAAGCGACAGCGGA	
*vgrG2*-E	CTAACTTGCACTTCCTCATCGTC	
*vgrG2*-F	CTTCAAGATCGTTCGTCTCC	
*sacB*-F	ACGGCACTGTCGCAAACTAT	Yu et al. (2012)
*sacB*-R	TTCCGTCACCGTCAAAGAT	

### HOMOLOGOUS RECOMBINATION

In-frame gene deletion of *vgrG2* was generated by *sacB*-based allelic exchange as described previously (Park et al., 2004; Yu et al., 2012). Briefly, PCR amplification was performed to generate the upstream and downstream fragments of the *vgrG2* gene (using respective primer pair vgrG2-A/B and vgrG2-C/D, **Table 2**). Overlap PCR was performed to construct a fragment with deletion of the *vgrG2* gene using the primer pair vgrG2-A/D. The fragment was cloned into pMD18T vector (Takara) and then subcloned into the suicide vector pYAK1 that contains the *sacB* gene conferring sensitivity to sucrose. The recombinant plasmid was introduced into *E. coli* CC118λpir and then mated with *V. parahaemolyticus* dTTT (strain HZ with in-frame deletion of *tdh*, *vcrD1*, and *vcrD2*) as the parent strain. The resulting mutant strains were screened using selective LB agar as specified above.

### PREPARATION OF PROTEIN SAMPLES FROM BACTERIAL SUPERNATANTS AND PELLETS

Secreted proteins from the parent and mutant *V. parahaemolyticus* strains were prepared from the supernatant samples of cultures grown for 16 h at 28^∘^C in LB broth. The samples were passed through a 0.2 μm pore-size syringe filter and precipitated by adding trichloroacetic acid to a final concentration of 10% (vol/vol). The proteins were collected by centrifugation at 15,000 *g* for 30 min at 4^∘^C. The precipitates were solubilized in 40 μl 0.1M NaOH, and 10 μl of 5x SDS-PAGE loading buffer was added prior to SDS-PAGE with 10% polyacrylamide. For separation of T6SS proteins associated with the bacterial cells, *V. parahaemolyticus* cultures were pelleted by centrifugation, and the pellets were resuspended in 10 mM phosphate buffered saline pH 7.2 (PBS, 100 mg wet weight pellet per ml). A volume of 160 μl was then mixed with 10 μl of 5X SDS-PAGE loading buffer, and the mixtures were heat-treated for 5 min in a boiling water-bath to release proteins from the bacterial cells before SDS-PAGE.

### CELLS CULTURE, BACTERIAL INFECTION, AND VECTOR TRANSFECTION

Murine RAW264.7 macrophage cells were cultured in Dulbecco’s modified Eagle’s medium (DMEM, Gibco) supplemented with 10% new-born calf serum, L-glutamine (1%), penicillin G (100 U/ml), and streptomycin (100 μg/ml).

The macrophage cells were infected with mid-log phase cultures (3–4 h) of *V. parahaemolyticus* wild-type strain (WT) HZ, and single or multiple deletion mutants at multiplicity of infection (MOI) of 10 at 37^∘^C and 5% CO_2_. Infection was allowed to proceed for 0.5 h and the supernatant was then removed. The cell monolayers were rinsed with sterile PBS to remove unattached bacteria and then incubated in the presence of fresh medium for 2 or 4 h at 37^∘^C and 5% CO_2_ for cytotoxicity assay or SDS-PAGE and immunoblotting as described below. Rapamycin treated (0.5 μM, Merck) or mock-infected cells were included as controls (here and in other relevant experiments).

The cells stably expressing GFP-LC3B on the coverslips were infected with the strain dTTT or dTTT-Δ*icmF2* at MOI of 10. The remaining procedure is the same as above. At the end of 4 h incubation, the cell monolayers were subjected for confocal microscopic imaging.

The macrophage cells were transfected with pcDNA-*egfp*, pHcp2, or pVgrG2 using lipofectamine 2000 (Invitrogen) for transient expression of the target proteins. The transfection medium was replaced 6 h post-transfection by complete medium containing G418 (0.5 mg/ml), and the cells were incubated for 24 h at 37^∘^C and 5% CO_2_ for analysis of LC3 lipidation by immunoblotting.

For examination of the effects of 3-methyladenine (3-MA) and chloroquinine (CQ) on autophagic response, the RAW264.7 cells were first treated with these agents (10 mM 3-MA, and 5 mM CQ) for 1 h before they were transfected with vectors pHcp2, pVgrG2, and pcDNA-*gfp* as described above. The cell pellets were then collected for SDS-PAGE/Western blotting for LC3-II and β-actin.

### ANALYSIS OF cAMP IN MACROPHAGES INFECTED WITH *Vibrio parahaemolyticus* STRAINS

For measurement of cAMP within macrophages infected with the bacterial strains, the RAW264.7 cell monolayers were infected with the parent and mutant strains (triple deletion mutant dTTT with additional deletion of *hcp2* or *vgrG2*, namely dTTT-Δ*hcp2* or dTTT-Δ*vgrG2*; about 10 MOI). After 4 h of incubation at 37^∘^C and 5% CO_2_, the cell monolayers were washed twice with PBS and lysed with deionized H_2_O for 10 min followed by repeated pipetting and vortex-mixing in Eppendorf tubes. The cell lysates were subjected to centrifugation as above and the supernatant samples were collected for analysis of intracellular cAMP using the ELISA-based cAMP assay kit (detection range from 0.5 to 6 nM, lot No HL30028, Shanghai Haling Biol. Technol. Co., Ltd., China) according to the manufacturer’s instruction.

### SDS-PAGE AND IMMUNOBLOTTING

After infection or transfection for indicated times, all cell samples were lysed for 10 min in ice cold lysis buffer [50 mM Tris-HCl pH 8.0, 140 mM NaCl, 1.5 mM MgCl_2_, 0.5% NP-40 with complete protease inhibitor cocktail (Roche)]. Cell debris was pelleted by centrifugation and clear supernatants transferred to new tubes. Protein concentration was measured by BCA protein assay kit (MultiSciences, Hangzhou, China). Protein samples were boiled for 5 min in the presence of 5x SDS-PAGE loading buffer.

Proteins on the gels were electro-transferred onto an Immobilon-P membrane (Millipore). The membranes were blocked with 5% skim milk in tris-buffered saline (20 mM Tris, 137 mM NaCl, pH 7.6) containing 0.05% Tween 20 and probed for 1 h with rabbit anti-Hcp2 or rabbit anti-VgrG2 polyclonal antibodies (Yu et al., 2012), rabbit anti-LC3 polyclonal IgG (Sigma–Aldrich) or anti-β-actin monoclonal IgG (MultiSciences, Hangzhou, China) for 3 h at room temperature. The blots were then probed with goat anti-rabbit or anti-mouse horse-radish peroxidase-labeled antibodies (KPL), and developed by the SuperSignala West Pico Chemiluminescent Substrate (Pierce) according to the manufacturer’s instruction.

### CONFOCAL MICROSCOPY

Cells infected as above were washed with PBS, fixed and permeabilized with 80% cold acetone in PBS at -20^∘^C for 20 min, and washed again with PBS. Cells were then counterstained with 4^′^,6-diamidino-2-phenylindole (DAPI) nucleic acid stain (Invitrogen). Fluorescence was observed under a laser scanning confocal microscope (Leica TCS SP5, Munich, Germany). The average number of EGFP-LC3 punctae per cell from at least 60 cells per sample was counted (Mizushima et al., 2010).

### CYTOTOXICITY ASSAY

The culture supernatants from infected macrophage cells as above were collected for lactate dehydrogenase activity (LDH) using CytoTox 96 non-radioactive cytotoxicity assay (Promega).

### STATISTICAL ANALYSIS

All data, where appropriate, are mean ± SD from three independent experiments and analyzed by Student’s *t*-test.

## RESULTS

### T6SS2 INDUCES AUTOPHAGY IN MACROPHAGE CELLS

**FIGURE 1:**
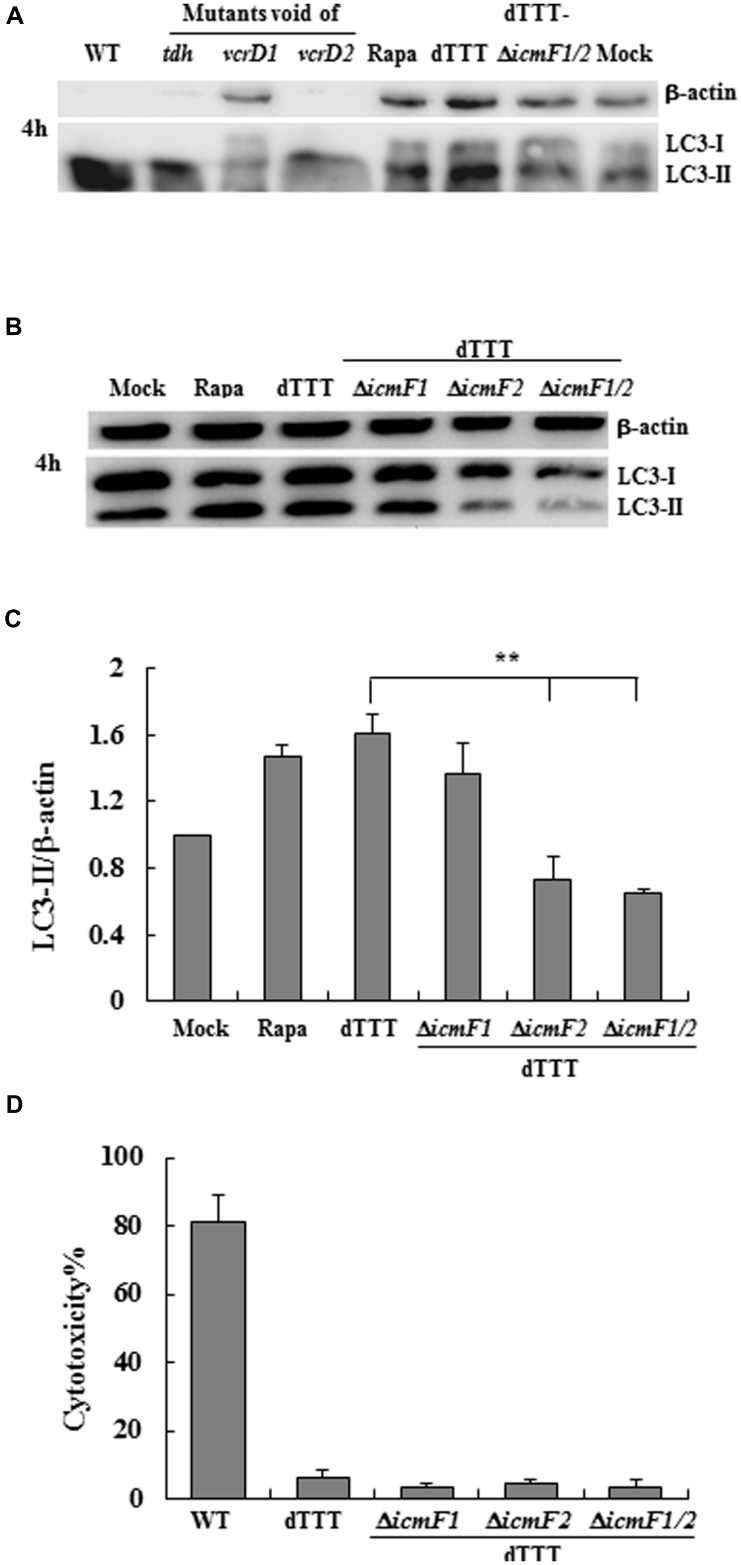
***Vibrio parahaemolyticus* T6SS2 induces autophagy of macrophage cells without causing apparent cytotoxicity. (A,B)** Autophagy of macrophage cells was measured by LC3-II accumulation after infection for 4 h with the wild-type strain (WT), its triple deletion mutant dTTT or dTTT with further deletion of T6SS1 and T6SS2 structural genes. Rapamycin-treated (Rapa) and mock-infected (Mock) cells were used as positive and negative controls. **(C)** LC3-II to β-actin ratio (mean ± SD) of three independent experiments as shown in **(B**; **P < 0.01). **(D)** Cytotoxicity to macrophages infected as above was measured by LDH release.

Our preliminary data indicate that there was no apparent autophagic response of the macrophage cells in 2-h infection with *V. parahaemolyticus*, either the WT strain or its triple deletion mutant strain dTTT void of *tdh*, *vcrD1,* and *vcrD2* with or without additional deletion of *icmF1* and *icmF2* (structural genes of VpT6SS1 and VpT6SS2, respectively; data now shown). When infection continued up to 4 h, T3SS1 was found to be involved in autophagy shown as reduced LC3-II transformation in the macrophage cells infected with Δ*vcrD1* deletion mutant (**Figure 1A**), as previously reported (Burdette et al., 2009a; Zhou et al., 2010). In cells infected with the WT strain or its mutants Δ*tdh* and Δ*vcrD1*, significant cytotoxic effect at 4 h was seen possibly due to T3SS1. To exclude the confounding effects of cytotoxicity, the triple deletion mutant (dTTT, in-frame deletion of *tdh*, *vcrD1,* and *vcrD2*) was used as the parent strain to examine the possible role of VpT6SS in autophagy after further deletion of both *icmF1* and *icmF2*.

**FIGURE 2:**
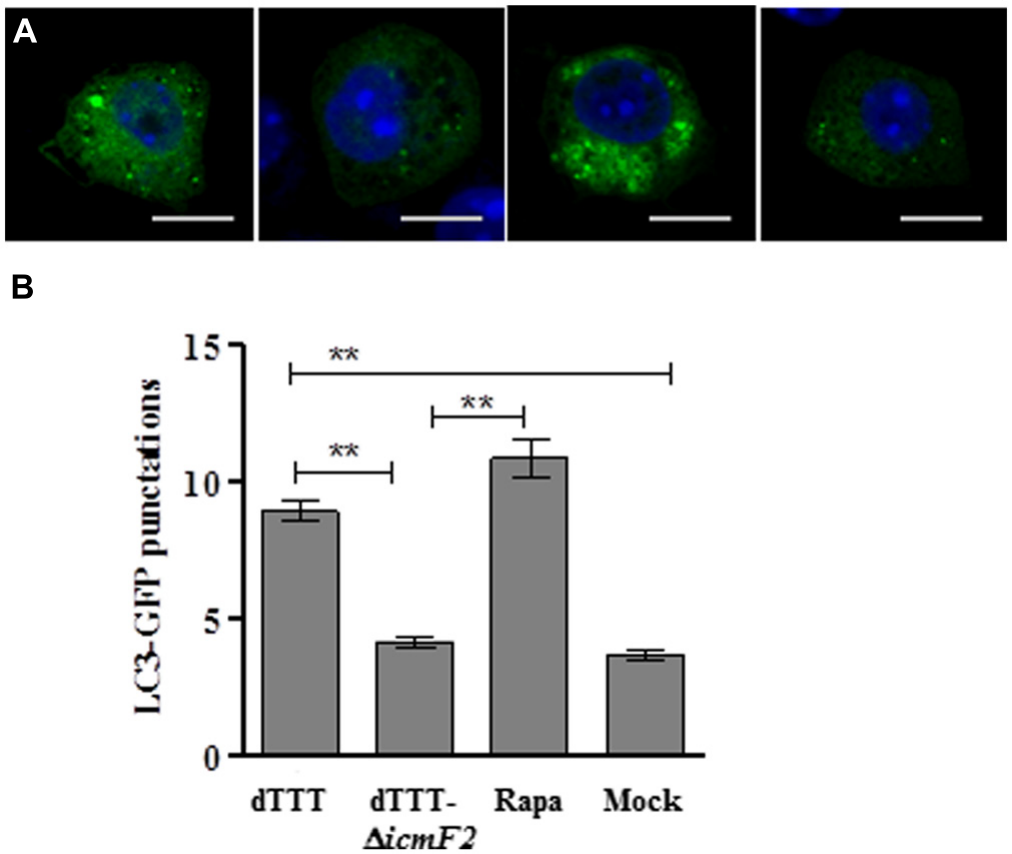
***Vibrio parahaemolyticus* T6SS2 induces autophagosome formation in macrophages. (A)** Formation of autophagosome vesicles shown as green punctae in EGFP-LC3 expressing macrophage cells infected with strain dTTT and its *icmF2* deletion mutant. Rapamycin-treated (Rapa) and mock-infected (Mock) cells were used as positive and negative controls (scale bar: 10 μm). **(B)** Mean ± SD of EGFP-LC3-II punctae per cell from 60 cells of three independent experiments as represented in (**A**; **P < 0.01).

Initial experiments with the multiple deletion strain did show reduced LC3 lipidation upon further deletion of both *icmF1 and icmF2* [dTTT-Δ*icmF1/2*, as compared with its parent strain dTTT or rapamycin positive control (**Figure 1A**)]. To explore which VpT6SS system triggered autophagy of macrophages, further deletion of *icmF1* or *icmF2* was attempted from the triple mutant strain dTTT. **Figures 1B,C** shows that LC3 lipidation was significantly reduced in dTTT-Δ*icmF1*/*2* or dTTT-Δ*icmF2*, but not dTTT-Δ*icmF1* (*P* < 0.01, as compared with its parent strain dTTT; **Figure 1C**), indicating that VpT6SS2 might be involved in the autophagic response. TDH and/or T3SS, but not VpT6SS contribute to cytotoxicity as revealed by lactate dehydrogenase release (**Figure 1D**). To further confirm whether VpT6SS2 was indeed related to autophagy and induce the formation of autophagosomes, the EGFP-LC3-expressing macrophage cells were infected with *V. parahaemolyticus* dTTT and its mutant dTTT-Δ*icmF2*. **Figure 2** shows that the mutant dTTT-Δ*icmF2* exhibited significantly reduced number of cells containing punctae of EGFP-LC3 (i.e., autophagosome-like vesicles), as compared with its parent strain dTTT (*P* < 0.01).

### VgrG2 OF VpT6SS2 IS THE EFFECTOR PROTEIN DEVOTING TO AUTOPHAGY

**FIGURE 3:**
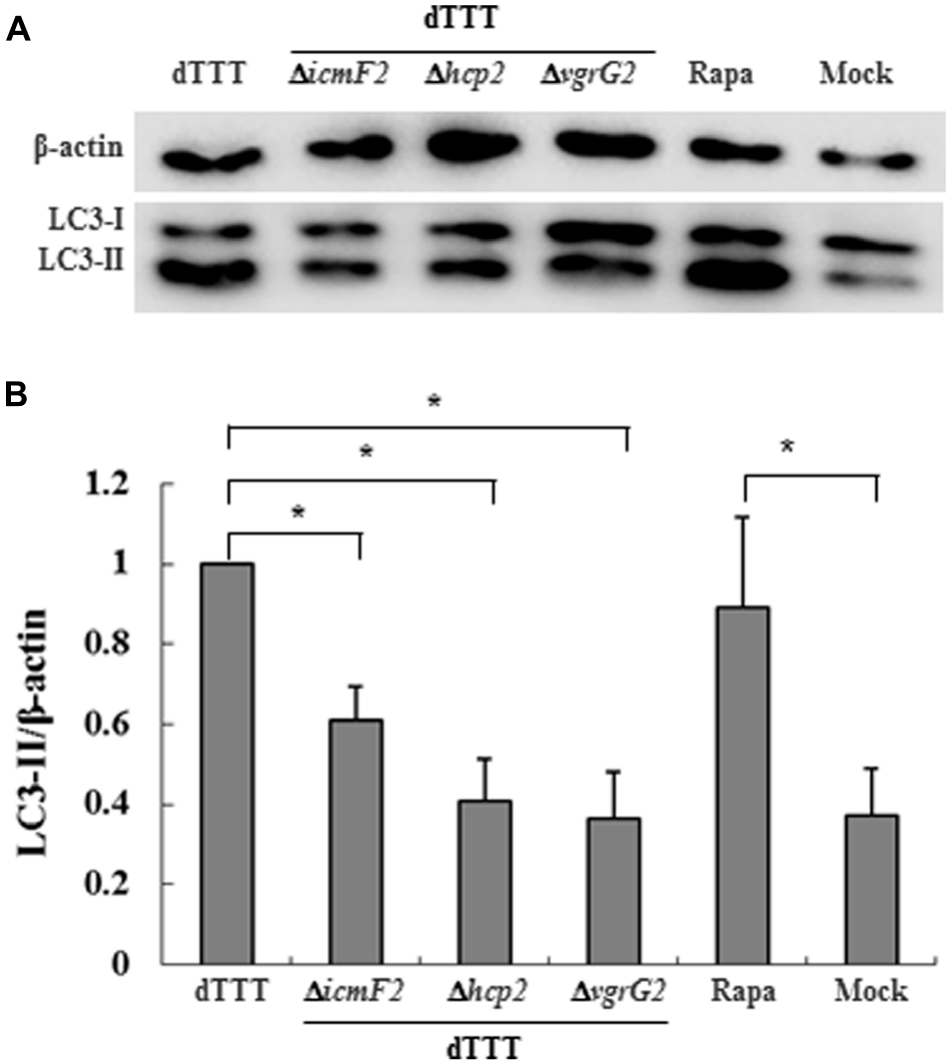
***Vibrio parahaemolyticus* T6SS2 translocons might be involved in autophagic response of macrophages. (A)** LC3-II accumulation in cells infected for 4 h with strain dTTT and its mutants. Rapamycin-treated (Rapa) and mock-infected (Mock) cells were used as positive and negative controls. **(B)** Ratios of LC3-II to β-actin were calculated from ‘**A**’ and reported as mean ± SD of three independent experiments with the dTTT ratio normalized to 1.0 (*P < 0.05).

A number of Gram-negative bacteria use T6SS to infect eukaryotic cells by its effectors, translocon, or secretion proteins (Cascales, 2008; Pukatzki et al., 2009). We paid attention to Hcp2 and VgrG2, two known translocon family proteins of VpT6SS2 (Yu et al., 2012). Deletion of either *hcp2* or *vgrG2* decreased the ratio of LC3-II to β-actin in cells infected with the mutant strains in comparison with its parent strain dTTT (**Figure 3**, *P* < 0.05), indicating that Hcp2, VgrG2, or even other secretion proteins translocated by them might contribute to autophagy in the macrophage cells.

**FIGURE 4:**
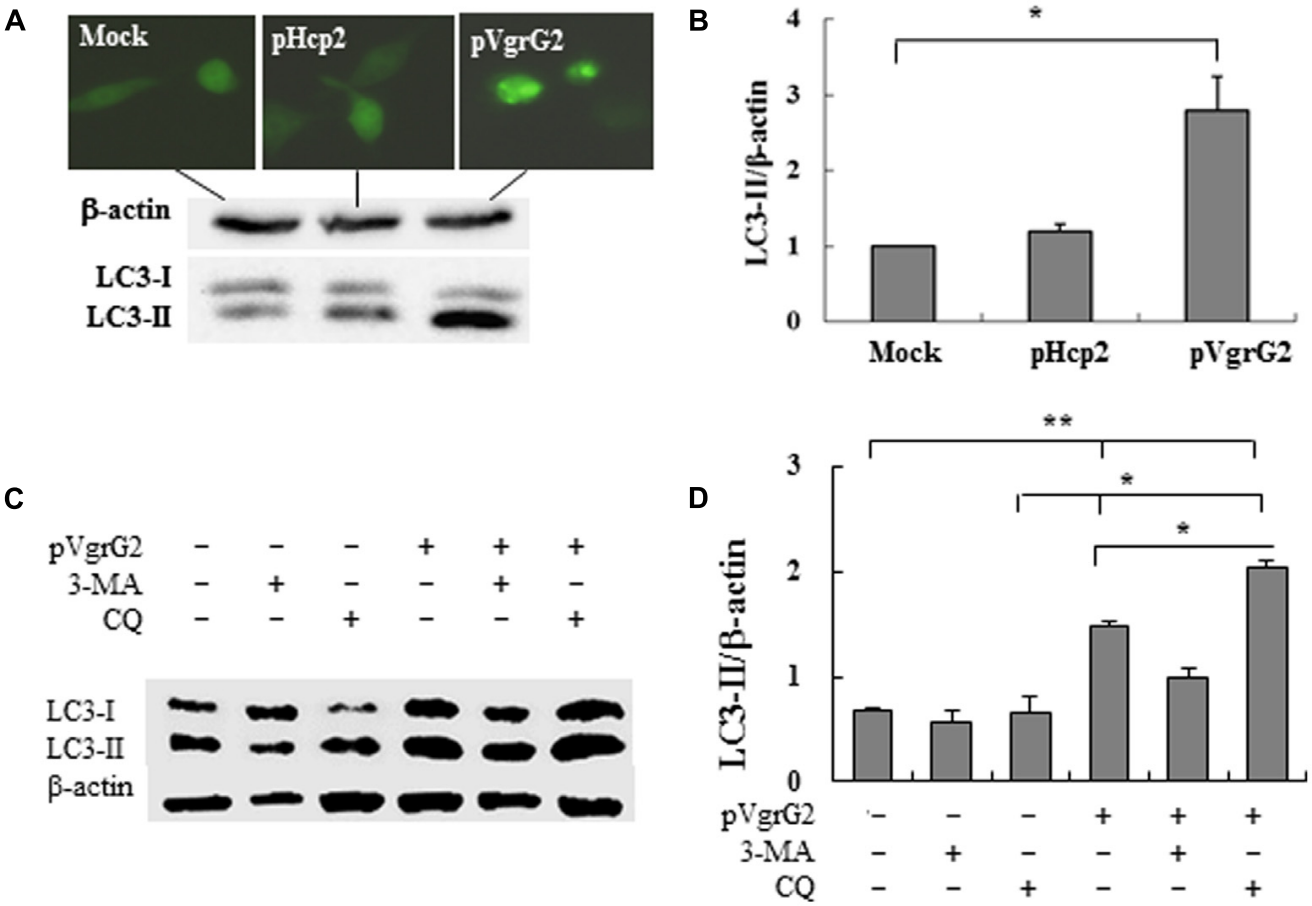
***Vibrio parahaemolyticus* T6SS2 VgrG2 is the effector protein inducing autophagy of macrophages. (A)** Hcp2 and VgrG2 protein were expressed in macrophage cells as the EGFP fusion proteins. LC3-II accumulation was measured by immunoblotting. **(B)** Ratios of LC3-II to β-actin of ‘**A**’ were calculated and reported as mean ± SD of three independent experiments with the ratio of Mock cells (transfected with the control vector pcDNA-*egfp*) normalized to 1.0. **(C)** Macrophage cells were pretreated for 1 h with 3-methyl adenine (3-MA) or chloroquinine (CQ) and then transfected with pVgrG2 or pcDNA-*egfp* for analysis of LC3-II by Western blotting. **(D)** Ratios of LC3-II to β-actin were calculated and reported as mean ± SEM of three independent experiments. *P < 0.05; **P < 0.01.

To further examine if Hcp2 or VgrG2 of VpT6SS devotes to autophagy, plasmids pHcp2, and pVgrG2 were made based on pcDNA-*egfp* (Zhu et al., 2012) to express EGFP-Hcp2 and EGFP-VgrG2 in macrophage cells after transfection for 24 h. We found that VgrG2, but not Hcp2, increased the conversion from LC3-I to LC3-II with significant elevation of LC3-II/β-actin ratio (*P* < 0.05) in comparison with the mock pcDNA-*egfp* (**Figures 4A,B**).

RAW264.7 cells were pretreated with P13-kinase inhibitors 3-MA and lysosome-phagosome fusion inhibitors chloroquinine. 3-MA is competent in its ability to inhibit induction of autophagy by rapamycin, a TOR kinase inhibitor, and well-characterized inducer of autophagy (Burdette et al., 2009a). The VgrG2-induced autophagy appears to be dependent of PI3-kinase activation because treatment with 3-MA repressed VgrG2-induced LC3-II accumulation (**Figures 4C,D**). Chloroquinine is a inhibitor of lysosome–phagosome fusion, which blocks degradation of LC3-II (Menzies et al., 2012). Chloroquinine treatment increased LC3-II lipidation in VgrG2-expressing cells, suggesting that VgrG2 enhanced the autophagic flux. These results indicate that VgrG2 of *V. parahaemolyticus* induces autophagy by targeting the initial events of autophagic signaling.

**FIGURE 5:**
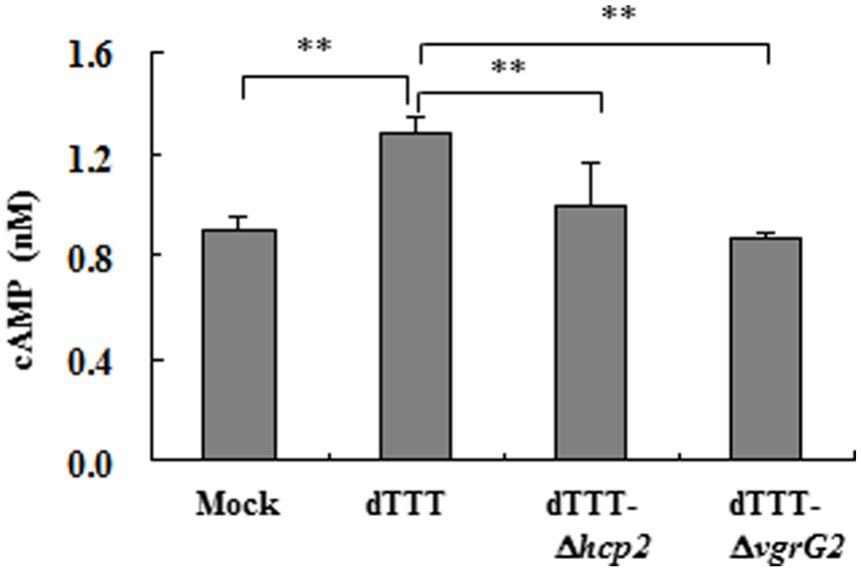
**Intracellular cAMP of macrophages infected with *V. parahaemolyticus* vgrG2 deletion mutant was significantly reduced as compared with its parent triple deletion strain dTTT.** The confluent RAW264.7 cell monolayers were infected with bacterial strains, each in triplicate wells, for 4 h. The culture supernatants were then removed, and the cell monolayers washed and lysed for cAMP determination. Each datum point represents mean ± SD of triplicate wells (**P < 0.01).

Besides, Cyclic AMP is known to activate AMPK which might contribute to autophagy due to repression of mTOR (Shaw, 2009). **Figure 5** indicates that the ΔvgrG2 mutant infected cells showed similar level of intracellular cAMP to the mock-infected cells, but had significantly lower level of intracellular cAMP than its parent strain, suggesting that *V. parahaemolyticus* vgrG2 might activate the cAMP signaling pathway to induce the autophagic response.

## DISCUSSION

Bacterial T6SS is a newly found secretion system which could be involved in pathogenesis and environment adaptation (Schwarz et al., 2010). Our previous study reveals that both T6SS1 and T6SS2 of *V. parahaemolyticus* contribute to adherence to mammalian cells (Yu et al., 2012). Here, we further show that T6SS2 of *V. parahaemolyticus* is involved in autophagic response of macrophages RAW264.7.

*Vibrio parahaemolyticus* contains several cytotoxic factors such as TDH and effector proteins of T3SS1 and T3SS2 which disrupt mammalian cell structure and lead to release of cellular contents (Burdette et al., 2008, 2009a; Zhou et al., 2010). In this study, we found the absence of β-actin bands and altered motility patterns of LC3 in cells infected with the WT, Δ*tdh* and Δ*vcrD2* strains, but not in the Δ*vcrD1* mutant. This might indicate that T3SS1 contributed to loss of β-actin as a result of acute autophagy accompanied with cell wall disruption and release of cellular contents, as shown in previous studies (Burdette et al., 2009a,b; Zhou et al., 2010). Recent studies show that two T3SS1 effectors, VopQ and Vp1659, contribute to autophagy in eukaryotic cells (Burdette et al., 2009a; Zhou et al., 2010). In order to examine autophagy induction by T6SS independent of other factors and to avoid interference by cytotoxic effects, we constructed T6SS deletion mutants based on the triple deletion mutant void of *tdh*, *vcrD1,* and *vcrD2* (strain dTTT) as the parent strain. It is clear that the triple deletion mutant dTTT and the mutant strains having further deletions of T6SS genes only showed marginal cytotoxicity as shown by LDH release.

Initially, we found that the multiple deletion mutant dTTT-Δ*icmF2*, but not dTTT-Δ*icmF1*, reduced autophagic response upon 4 h of infection of the macrophage, suggesting involvement of the putative T6SS2. Since *icmF1* or *icmF2* is the inner membrane protein of T6SS (Cascales, 2008), we attempted to search for the possible effector proteins that could be responsible for direct induction of autophagy by further deletion of the genes encoding Hcp2 and VgrG2, two putative translocons of T6SS2 seen in several bacterial species (Cascales, 2008) and also in T6SS2 of *V. parahaemolyticus* according to our previous study (Yu et al., 2012). Deletion of either *hcp2* or *vgrG2* did lessen the autophagic response as shown by decreased ratio of LC3-II to β-actin. However, increased LC3-II lipidation was seen only in the macrophage cells transfected with pVgrG2, but not with pHcp2. Therefore, we conclude that VgrG2, but not Hcp2, is the autophagy inducer of *V. parahaemolyticus*. Hcp might form as the tubular structure of VpT6SS for effector proteins including VgrG to translocate across the bacterial cell wall (Cascales and Cambillau, 2012). Deletion of *hcp2* could apparently prevent VgrG2 translocation, leading to decreased LC3-II.

The fact that chloroquinine treatment caused accumulation of LC3-II indicates that VgrG2 could probably act on the upstream autophagic pathways. We also provide evidence that the cAMP signaling might be involved in autophagy induction by VgrG2 of *V. parahaemolyticus* since *vgrG2* deletion led to reduced level of intracellular cAMP as compared with its parent strain. Different pathogens might explore distinct mechanisms in autophagic responses. *Staphylococcus aureus* was reported to enhance non-canonical autophagic response by its α-hemolysin in a PI3K/Beclin1-independent way, which is inhibitable by treating the cells with a permeable dibutyryl cAMP (a cAMP analog; Mestre and Colombo, 2012). They found that such inhibition was related to recruitment of Epac (Rap guanine nucleotide exchange factor/exchange protein activated by cAMP) and Rap2b via calpain activation. However, Chen et al. (2013) show that cAMP was necessary for resveratrol-induced PRKA-AMPK-SIRT1 activation of autophagy in human umbilical vein endothelial cells treated with this naturally occurring phytoalexin compound). Therefore, the possible mechanisms of cAMP signaling in *V. parahaemolyticus* VgrG2 induced autophagy require further investigation.

In conclusion, *V. parahaemolyticus* is clearly equipped with two arms of autophagy inducers, T3SS1 and T6SS2. However, T6SS2 induces autophagy without involving cytotoxic effect as seen in T3SS1.

## Conflict of Interest Statement

The authors declare that the research was conducted in the absence of any commercial or financial relationships that could be construed as a potential conflict of interest.
